# Supplementary material for: Restrictive versus liberal fluid resuscitation strategy, influence on blood loss and hemostatic parameters in mild obstetric hemorrhage: An open-label randomized controlled trial. (REFILL study)
Source: PLoS One. 2021 Jun 25;16(6):e0253765. doi: 10.1371/journal.pone.0253765 (PMC8232446; doi:10.1371/journal.pone.0253765)
Supplement: S1 File — BMI: body mass index PPH: postpartum hemorrhage. (DOCX) [file pone.0253765.s002.docx]

**S1 File – Risk factors postpartum haemorrhage**

| **General information** |
| --- |
| Age (> 40 years, not multiparous) |
| Obesity (BMI >35) |
| Grand multipara |
| Uterus myomatosus |
| Other, If yes, please specify |
|  |
| **Obstetric history** |
| History of manual removal of placenta(l fragments) |
| History of PPH |
| Other, If yes, please specify |
|  |
| **Current pregnancy** |
| Anaemia (< 96·7g/L) |
| Chorioamnionitis |
| Gestational hypertension |
| Multiple pregnancy |
| Macrosomia |
| Placenta praevia/accrete |
| Other, If yes, please specify |
|  |
| **Current delivery** |
| Induction of labour |
| Augmentation of labour |
| Fever in labour |
| Prolonged first stage of labour (>10 hours) |
| Prolonged second stage of labour (> 60 minutes) |
| Suspected or proven placental abruption |
| Mediolateral episiotomy |
| Ventouse or forcipal extraction |
| Delivery by emergency caesarean section |
| Delivery by elective caesarean section |
| Retained placenta |
| Macrosomia (> 4 kg) |
| Other, If yes, please specify |

BMI: body mass index PPH: postpartum haemorrhage
